# Extensive Multiple Sclerosis Reactivation after Switching from Fingolimod to Rituximab

**DOI:** 10.1155/2018/5190794

**Published:** 2018-07-19

**Authors:** Trygve Holmøy, Øivind Torkildsen, Svetozar Zarnovicky

**Affiliations:** ^1^Department of Neurology, Akershus University Hospital, Lørenskog, Norway; ^2^University of Oslo, Oslo, Norway; ^3^Department of Neurology, Haukeland University Hospital, Bergen, Norway; ^4^Department of Clinical Medicine, University of Bergen, Bergen, Norway; ^5^Department of Radiology, Akershus University Hospital, Lørenskog, Norway

## Abstract

During treatment with fingolimod, B cells are redistributed from blood to secondary lymphoid organs, where they are protected from the effect of anti-CD20 and other cell-depleting therapies. We describe a multiple sclerosis patient who had almost complete depletion of B cells in blood during and shortly after treatment with fingolimod. He developed severe disease activity resembling immune reconstitution syndrome after switching from fingolimod to rituximab, with first dose being six weeks after fingolimod cessation. Following recommendations from the Swedish MS Association, rituximab treatment was started as one single dose of 1000 mg. In patients treated with fingolimod, pathogenic B cells may still be sequestered in secondary lymph nodes if this dose is given early. To deplete such B cells as they egress from the lymph nodes, we propose that a second dose of rituximab a few weeks after the first dose should be considered.

## 1. Introduction

Although data from patients who participated in randomized clinical trials suggest that disease activity returns to pretreatment levels in most multiple sclerosis (MS) patients who discontinue fingolimod [1], several case reports suggest that some patients experience unexpectedly severe disease activity consistent with a rebound phenomenon [2–6]. In one recent study unexpectedly severe disease reactivation was reported in all nine patients who switched from fingolimod to alemtuzumab, possibly because fingolimod induces prolonged sequestration of pathogenic lymphocytes in secondary lymphoid organs where they are protected from killing by alemtuzumab [7]. Fingolimod has profound effects on B cell numbers and composition in blood [8], including memory B cells which are the main targets of anti-CD20 therapies like rituximab and ocrelizumab [9]. Prolonged retention of B cells after cessation of fingolimod may have consequences for the effect of subsequent B cell directed therapies. We report a patient with severe MS exacerbation resembling immunological reconstitution syndrome (IRIS) after switching from fingolimod to rituximab, another cell-depleting monoclonal antibody. The patient provided written informed consent to the publication of this report.

## 2. Case History

A 40-year-old man was diagnosed with MS in July 2011, after attacks with ataxia in December and March 2010 and numbness in both legs and right arm in May 2011. MRI revealed 50 T2 lesions in the brain, brainstem, and spinal cord and 12 contrast enhancing lesions in the cerebral hemispheres. He started natalizumab and was clinically and radiologically stable with Expanded Disability Status Scale (EDSS) score at 2.5 until January 2014, when he switched to fingolimod because a test for antibodies against John Cunningham virus was positive. MRI revealed several new T2 lesions in the right cerebral hemisphere in October 2015. In May 2016 he had an attack with vertigo and ataxia, and he had new contrast enhancing lesions in the left cerebral hemisphere and the right cerebellar hemisphere in September 2016.

After an application for hematopoietic stem cell therapy was rejected, switching treatment to rituximab was decided. At fingolimod discontinuation in May 2017, he had modest MS symptoms (EDSS 2.5) and was working 50% as senior project manager. Total lymphocyte count one week after discontinuation of fingolimod was 0.8x10^9^/l, with CD19 B cells at the lower detection limit (0.02 x10^9^/l; reference 0.1-0.5 x10^9^/l). CD4 T cells were less profoundly depressed (0.15 x10^9^/l; reference 0.3-1.4 x10^9^/l), and the numbers of CD8 T cells and CD56 NK cells were normal. At rituximab infusion (1000 mg) six weeks after discontinuation of fingolimod, total lymphocyte count normalized (1.4 x10^9^/l, subsets not counted).

**Figure 1:**
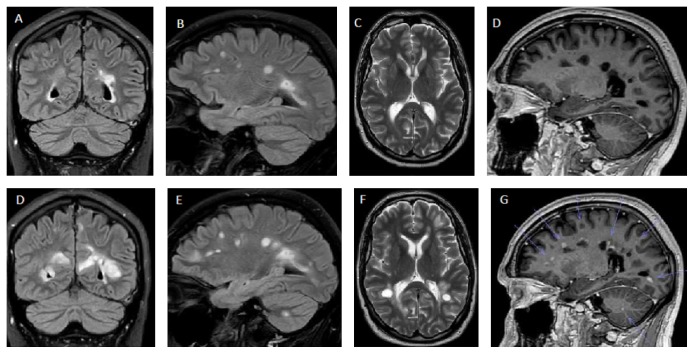
MRI before cessation of fingolimod in September 2016 (upper row) and 19 days after infusion of rituximab (July 2017; lower row). Coronal (**A/D **) and sagital (B/E) flair and coronal T2 (C/F) images show several new and enlarging lesions after switch to rituximab. Arrows indicate contrast enhancement on sagital T1 images (**D/G**).

The patient was hospitalized 19 days after rituximab infusion with rapidly evolving gait difficulties, diplopia, left hemiparesis, and altered mentation with pronounced irritability (EDSS 5.0). MRI showed approximately 50 contrast enhancing lesions throughout the cerebral hemispheres, mesencephalon, right upper and lower cerebellar peduncles, left cerebellar hemisphere, and corresponding to C3 and C5 in the spinal cord (Figure 1). He received 1000 mg methylprednisolone for five days followed by prednisolone for two months. After five weeks there were no new MRI lesions and no contrast enhancement. Another month later he was able to work 20%, but had bilateral diplopia and nystagmus, slight left sided hemiparesis, brisk tendon reflexes, and positive Babinski (EDSS at 3.5). He later remained clinically stable for another eight months.

Six weeks after rituximab, infusion B cells were not measurable in blood (< 0.02 x10^9^/l). CD4 T cells were still low (0.12 x10^9^/l).

## 3. Discussion

This case history underlines the role of B cells in MS. Memory B cells are suggested to be a shared target for effective MS drugs, including fingolimod and rituximab [10]. Infusion of 1000 mg rituximab rapidly depletes all B cells from the circulation and reduces MRI activity already at four to 12 weeks [11]. Fingolimod also profoundly depletes total B cell numbers, reduces the proportion of memory B cells [12], and increases the proportion of regulatory B cells [8]. Whereas it has been shown that normalization of T cell subsets may take months [13], the kinetics of B cell reconstitution after cessation of fingolimod has not been studied. In vitro studies suggest that the capacity for T lymphocytes migration across the blood-brain-barrier is suppressed during treatment with fingolimod although the expression of *α*4*β*1 integrin is high [14], which may facilitate enhanced migration upon cessation of fingolimod.

As our patient had high disease activity before initiation of natalizumab in 2011, we aimed for a short washout of fingolimod. He did, however, have almost completely suppressed B cell numbers and also reduced CD4 T cells after cessation of fingolimod. When deciding to wait for six weeks, we considered both the possibility of excessive immunosuppression from overlapping effects, and the possibility that pathogenic B cells sequestered in the lymph nodes could escape from the effect of rituximab and later reach the circulation if rituximab was given too early. Moreover, our patient did not experience excessive disease activity during the previous eight-week washout of natalizumab.

We are using a dosing regimen for rituximab recommended by the Swedish MS Association, which, based on registered data of 822 patients, recommends an initial dose of 1000 mg followed by 500 or 1000 mg every six months [15]. Repeated dosing two to four weeks after the first infusion is sometimes used and is not associated with more serious side effects [15]. Repeated initial dosing may be a reasonable alternative in patients switching from fingolimod, as it allows for a longer period with sufficient concentrations of rituximab in the blood to kill off B cells egressing from secondary lymphoid organs.

## References

[1] Vermersch P., Radue EW., Putzki N., Ritter S., Merschhemke M., Freedman M. S. (2017). A comparison of multiple sclerosis disease activity after discontinuation of fingolimod and placebo, Multiple Sclerosis Journal Experimental. *Translational & Clinical*.

[2] Havla J. B., Pellkofer H. L., Meinl I., Gerdes L. A., Hohlfeld R., Kumpfel T. (2012). Rebound of disease activity after withdrawal of fingolimod (FTY720) treatment. *JAMA Neurology*.

[3] Forci B., Mariottini A., Mechi C., Massacesi L., Repice A. (2017). Disease reactivation following fingolimod withdrawal in multiple sclerosis: Two case reports. *Multiple Sclerosis and Related Disorders*.

[4] Hatcher S. E., Waubant E., Nourbakhsh B., Crabtree-Hartman E., Graves J. S. (2016). Rebound syndrome in patients with multiple sclerosis after cessation of fingolimod treatment. *JAMA Neurology*.

[5] Meinl I., Havla J., Hohlfeld R., Kümpfel T. (2017). Recurrence of disease activity during pregnancy after cessation of fingolimod in multiple sclerosis. *Multiple Sclerosis Journal*.

[6] Giordana M. T., Cavalla P., Uccelli A. (2018). Overexpression of sphingosine-1-phosphate receptors on reactive astrocytes drives neuropathology of multiple sclerosis rebound after fingolimod discontinuation. *Multiple Sclerosis Journal*.

[7] Willis M., Pearson O., Illes Z., Sejbaek T. (2017). An observational study of alemtuzumab following fingolimod for multiple sclerosis, Neurology. *Neuroimmunology & Neuroinflammation*.

[8] Grutzke B., Hucke S., Gross C. C., Herold M. W. (2015). Fingolimod treatment promotes regulatory phenotype and function of B cells. *Annals Clinical & Translational Neurology*.

[9] Greenfield A. L., Hauser S. L. (2018). B-cell Therapy for Multiple Sclerosis: Entering an era. *Annals of Neurology*.

[10] Baker D., Marta M., Pryce G., Giovannoni G., Schmierer K. (2017). Memory B Cells are Major Targets for Effective Immunotherapy in Relapsing Multiple Sclerosis. *EBioMedicine*.

[11] Hauser S. L., Waubant E., Arnold D. L. (2008). B-cell depletion with rituximab in relapsing-remitting multiple sclerosis. *The New England Journal of Medicine*.

[12] Blumenfeld S., Staun-Ram E., Miller A. (2016). Fingolimod therapy modulates circulating B cell composition, increases B regulatory subsets and production of IL-10 and TGF*β* in patients with Multiple Sclerosis. *Journal of Autoimmunity*.

[13] Ghadiri M., Fitz-Gerald L., Rezk A. (2017). Reconstitution of the peripheral immune repertoire following withdrawal of fingolimod. *Multiple Sclerosis Journal*.

[14] Mathias A., Perriot S., Canales M., Blatti C. (2017). Impaired T-cell migration to the CNS under fingolimod and dimethyl fumarate. *Neurology Neuroimmunology & Neuroinflammatuion*.

[15] Salzer J., Svenningsson R., Alping P. (2016). Rituximab in multiple sclerosis. *Neurology*.

